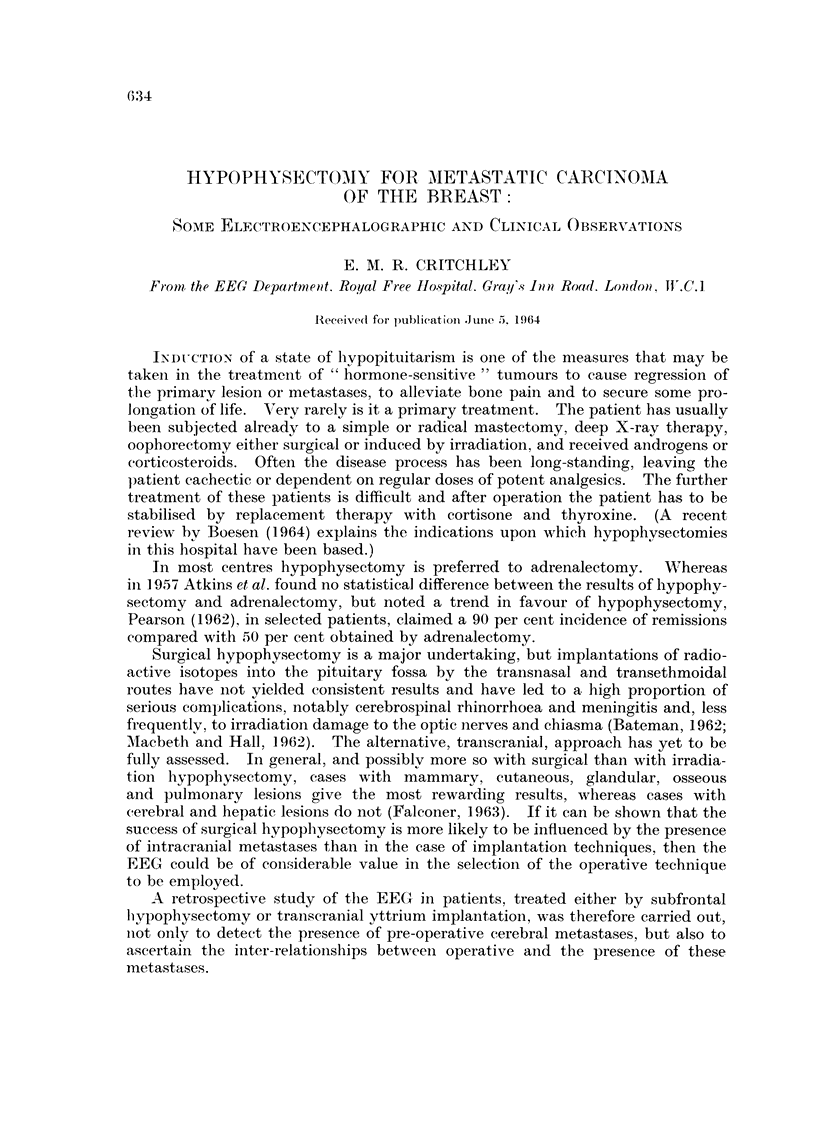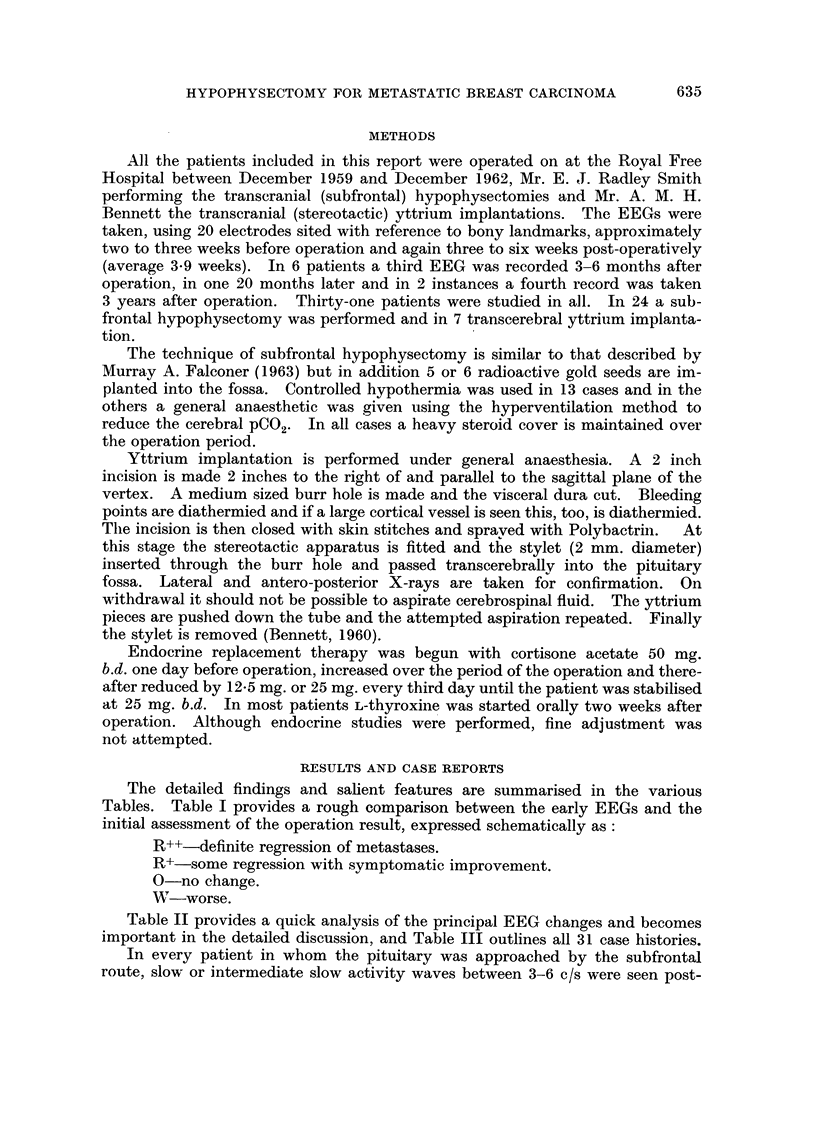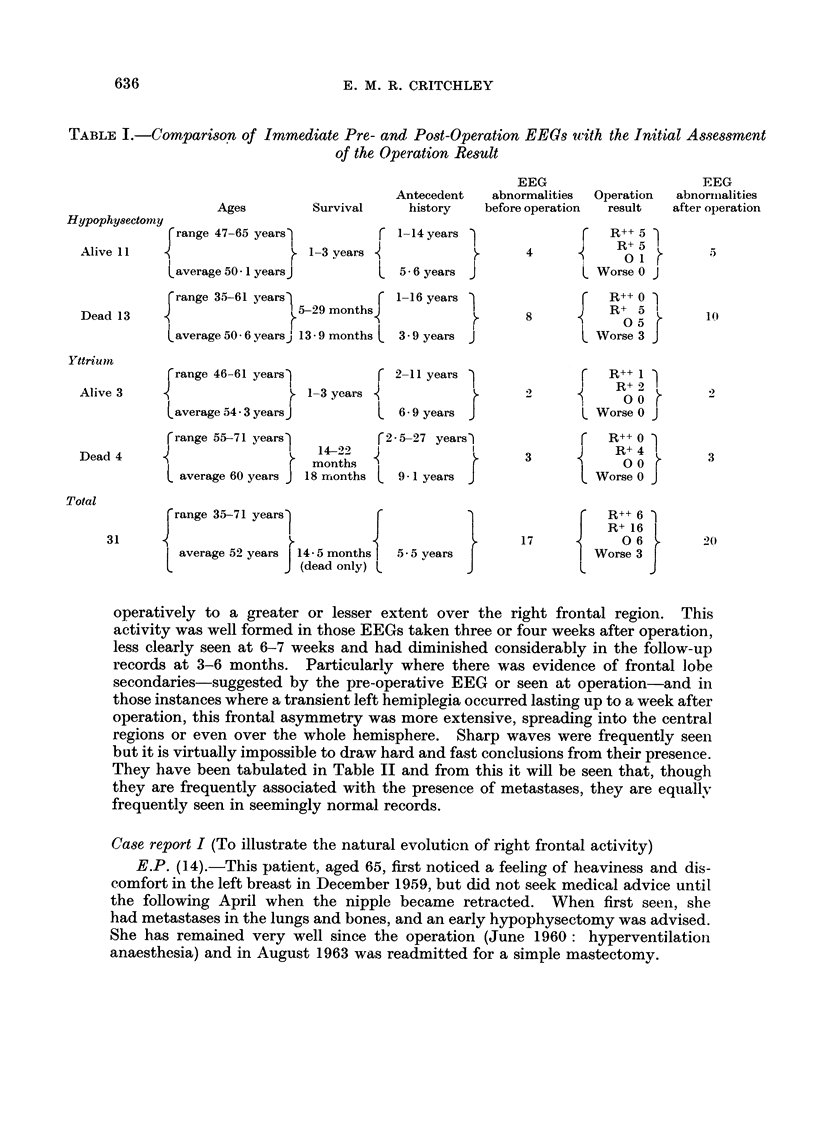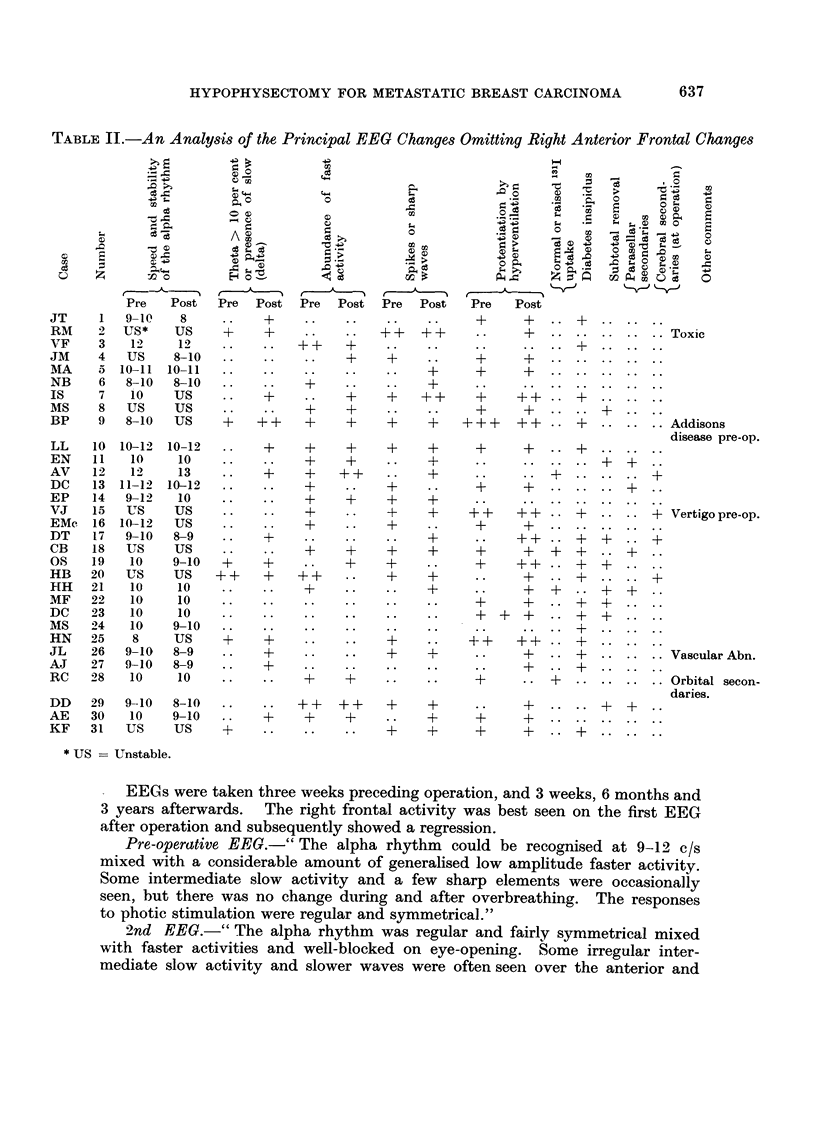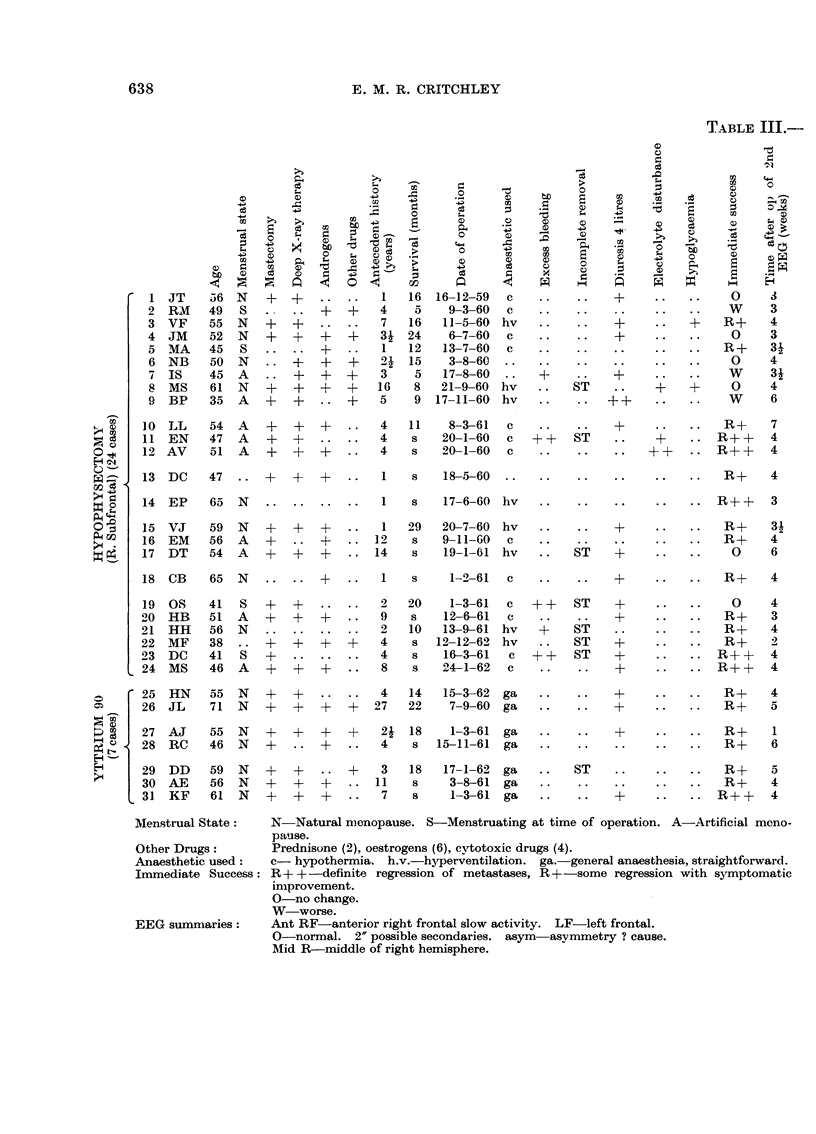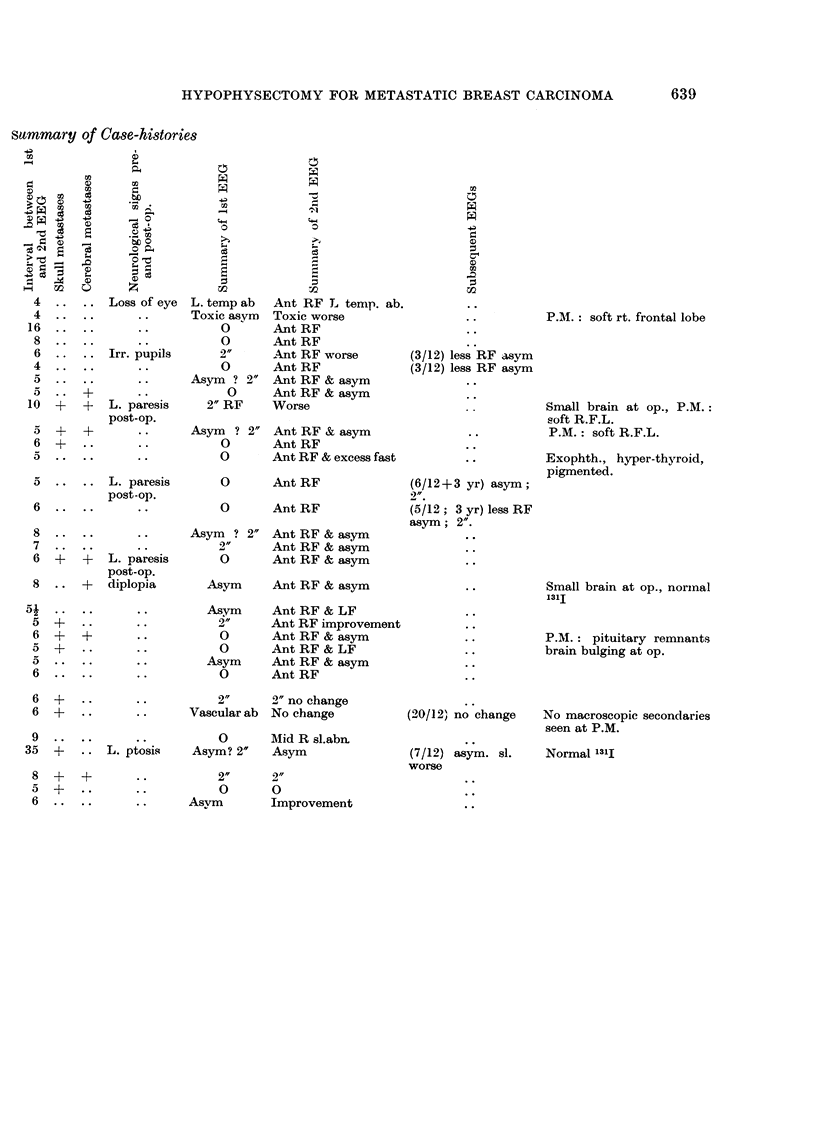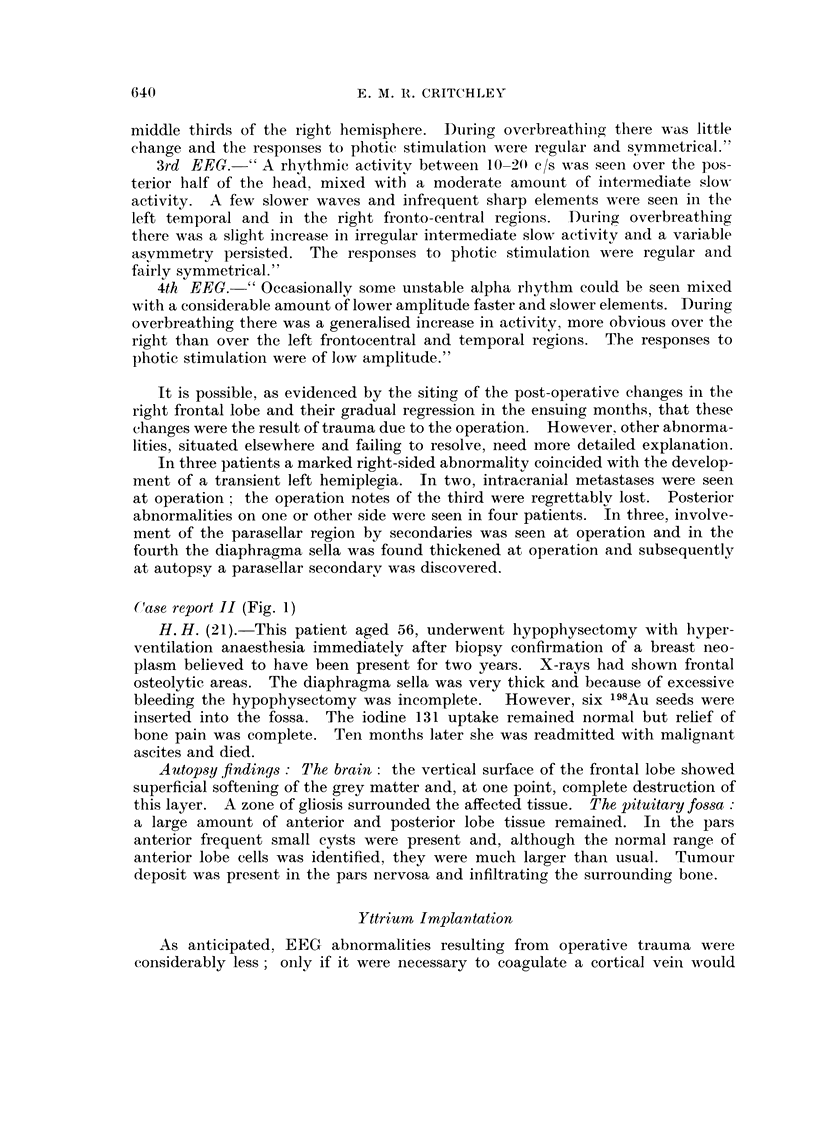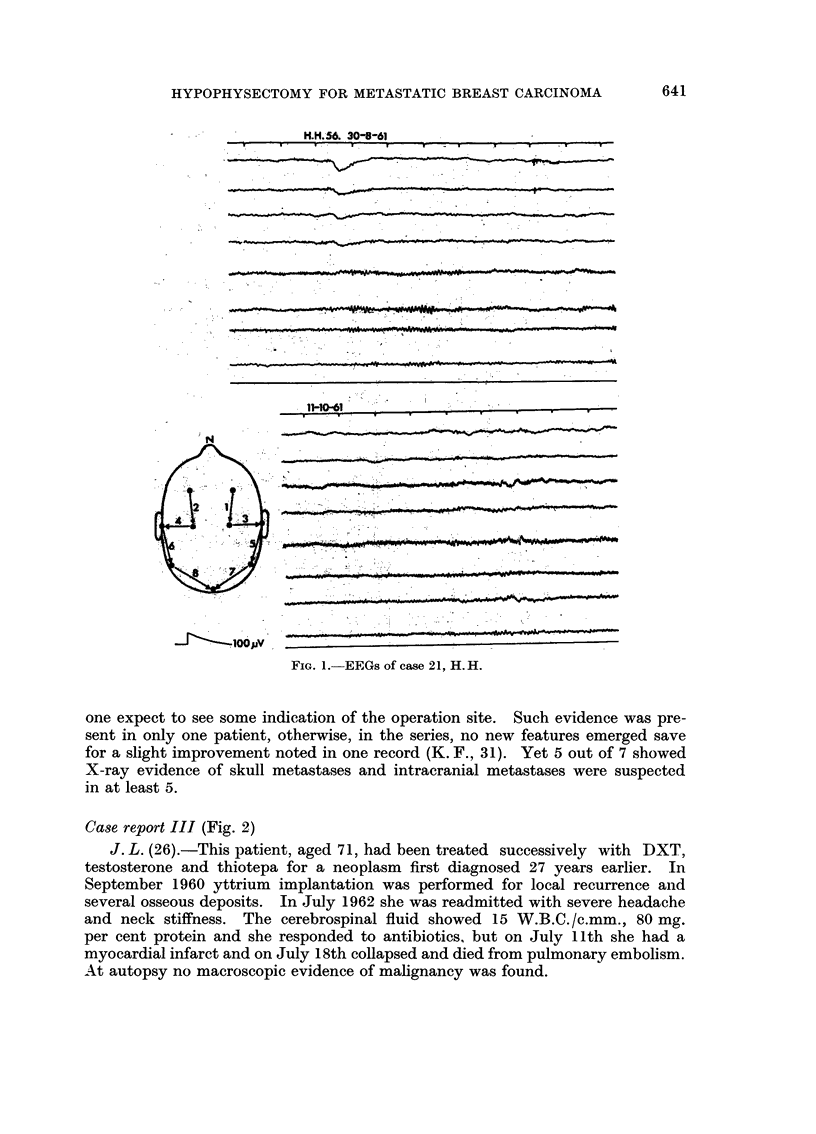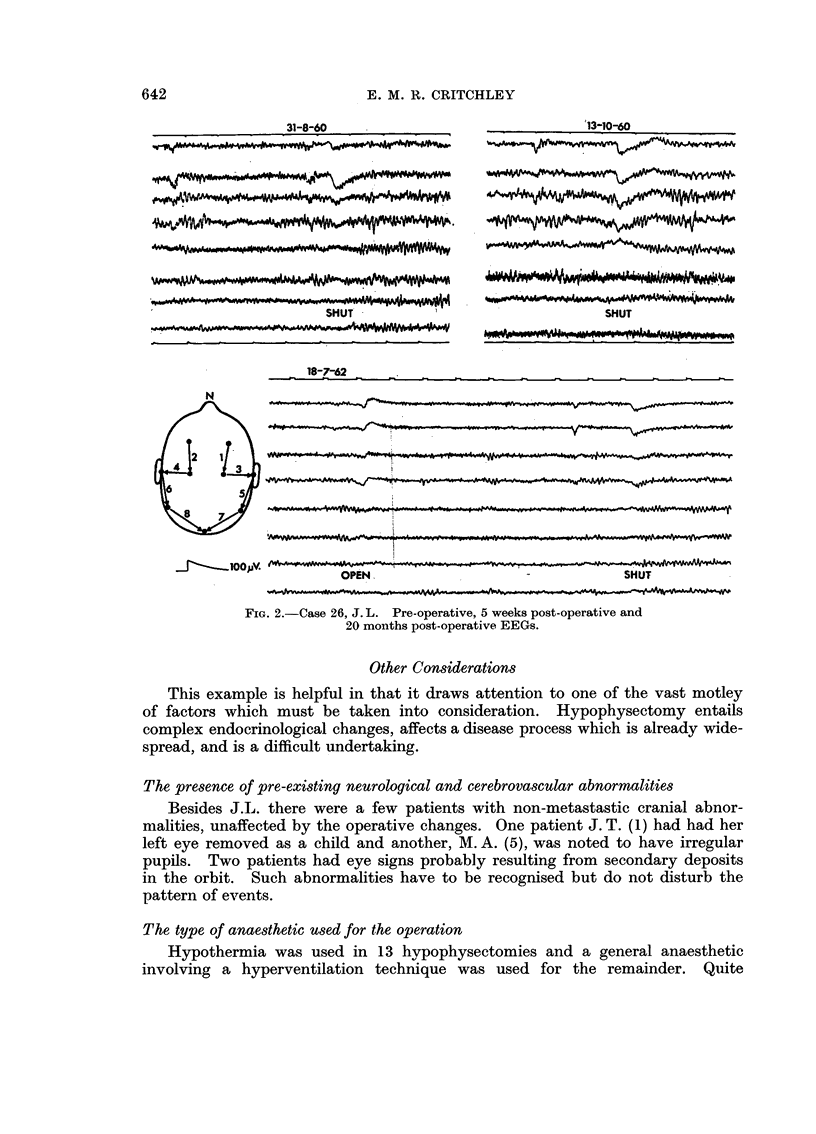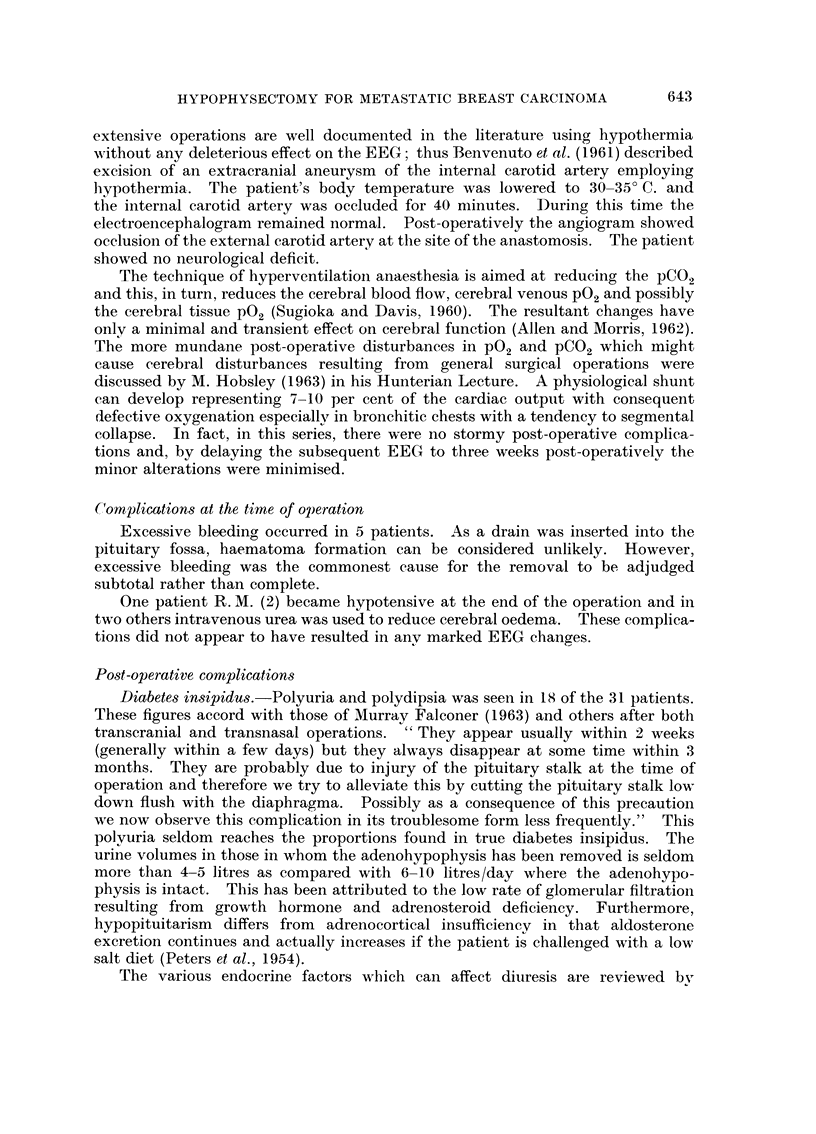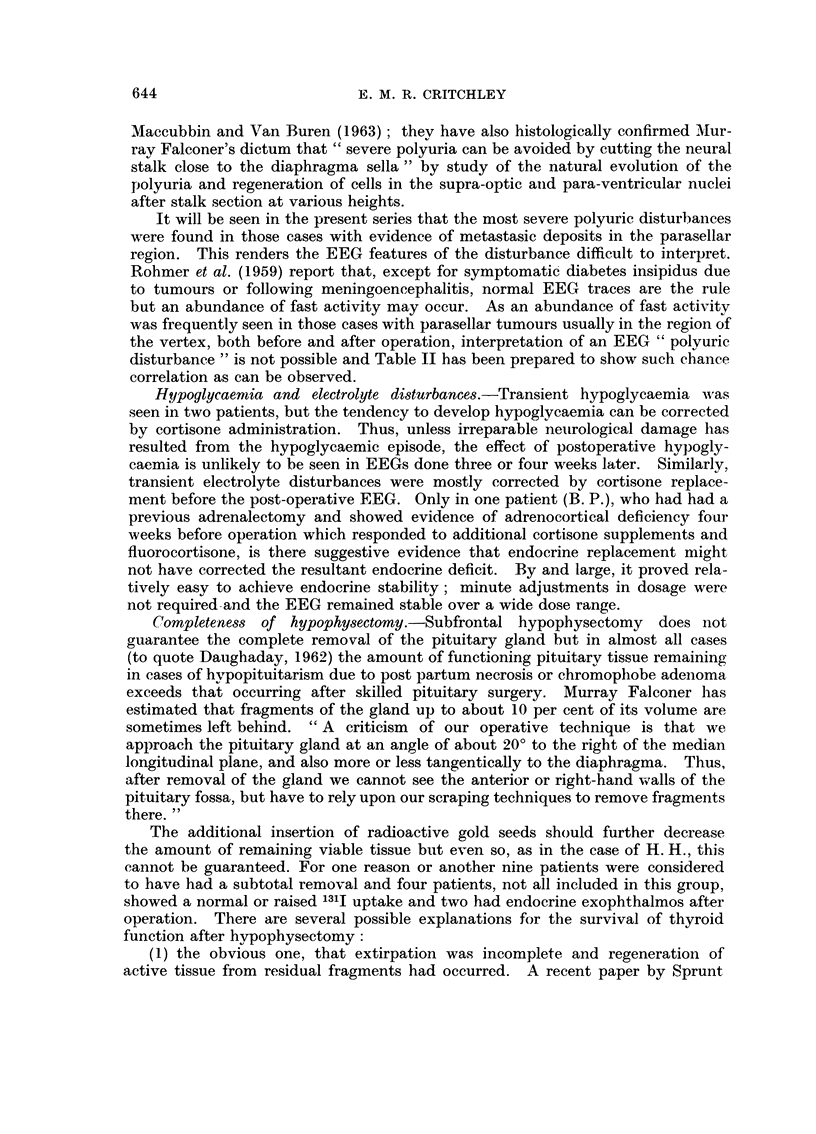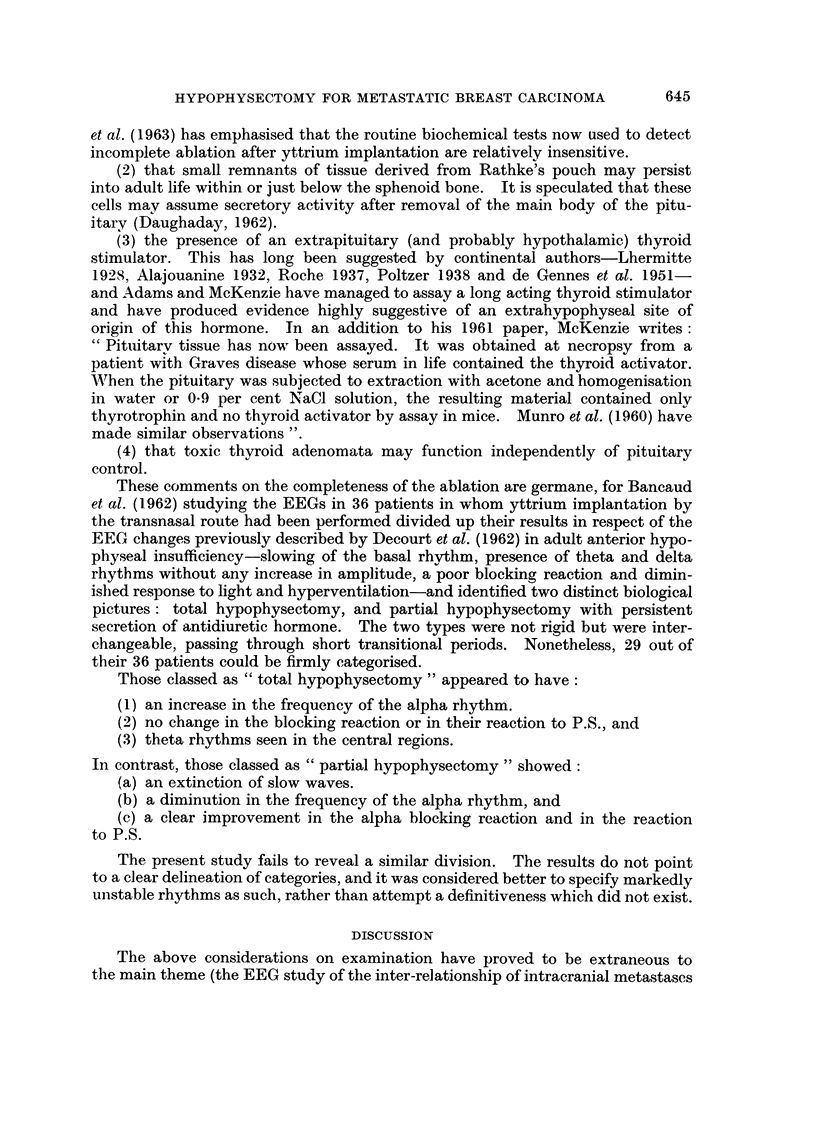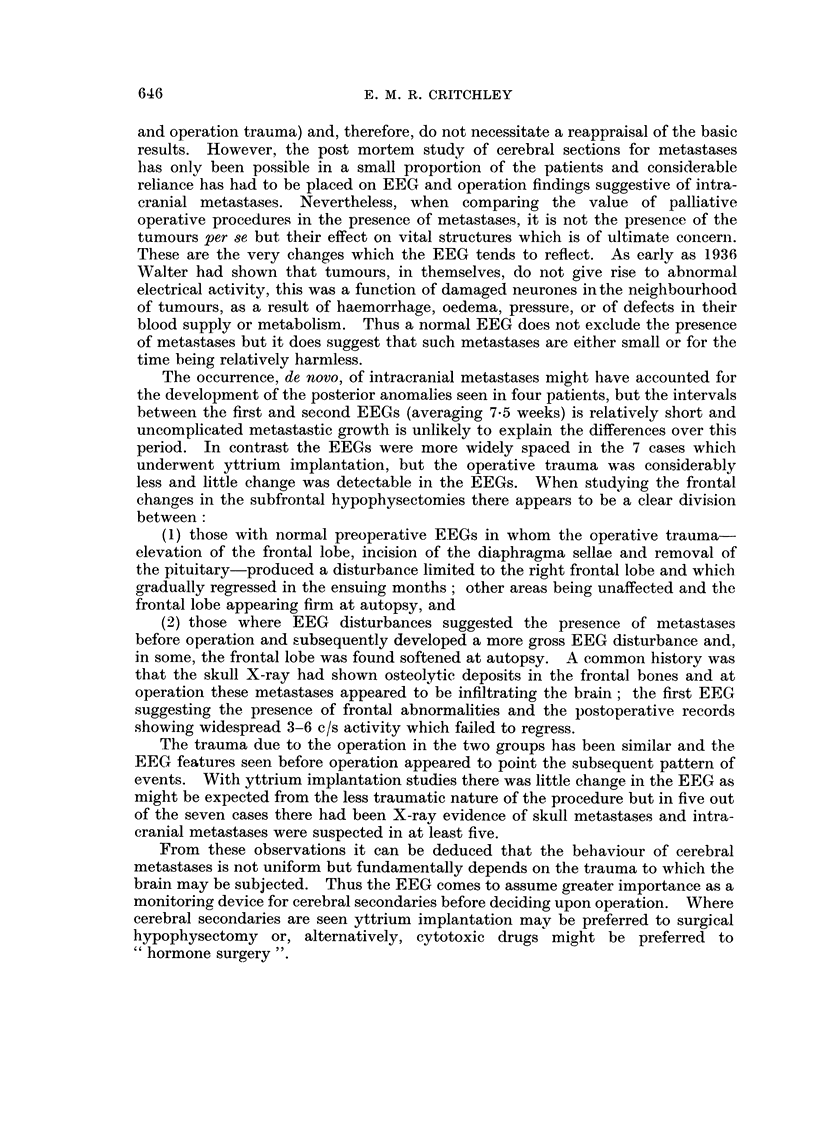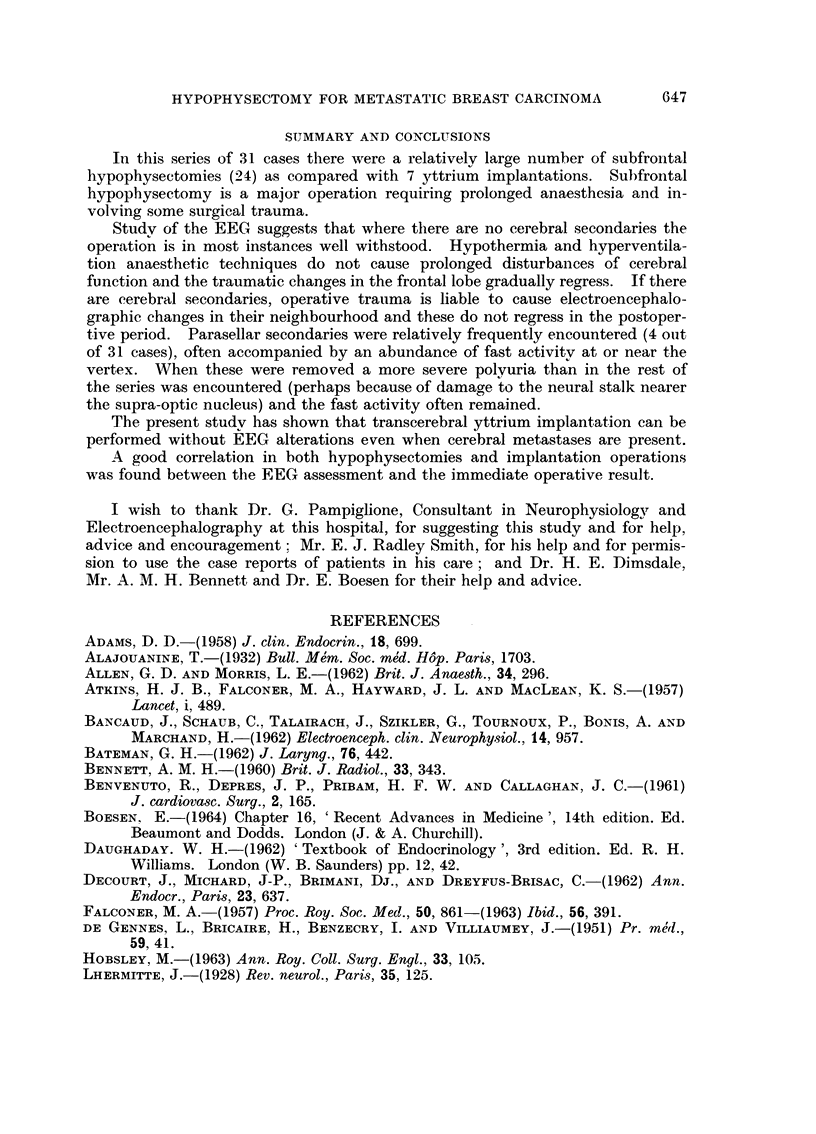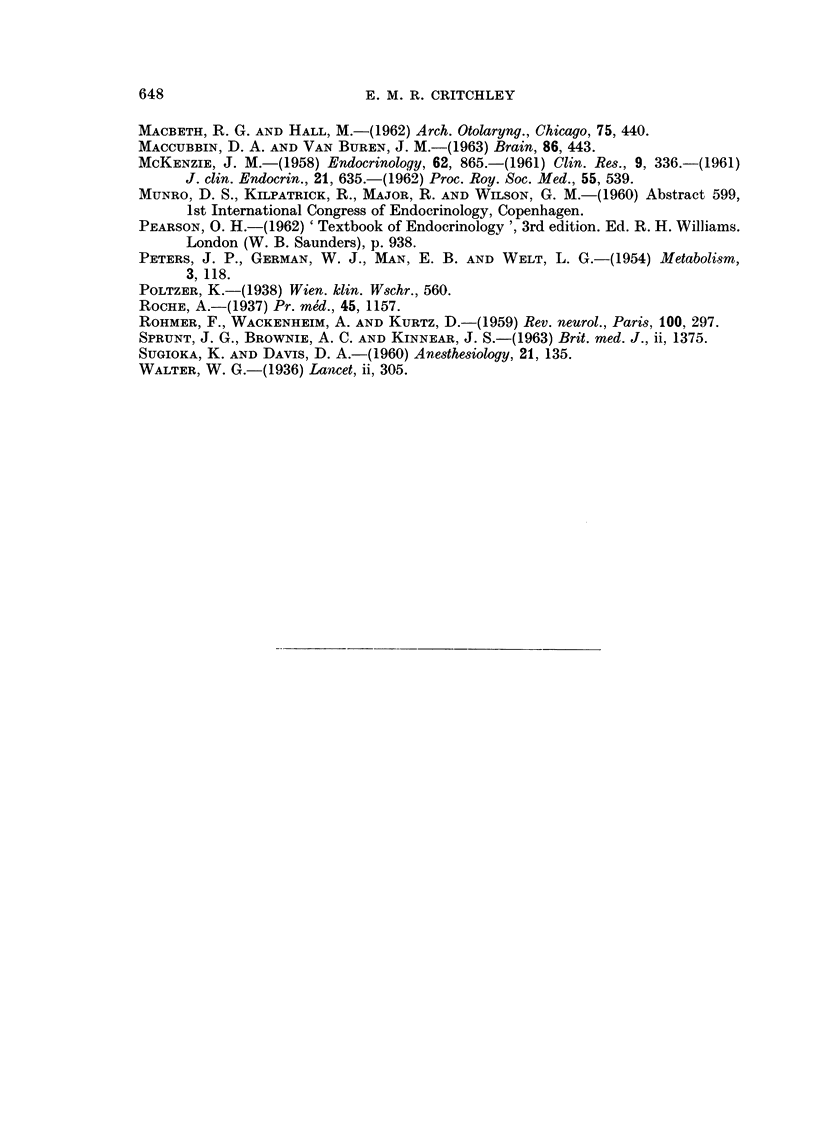# Hypophysectomy for Metastatic Carcinoma of the Breast

**DOI:** 10.1038/bjc.1964.73

**Published:** 1964-12

**Authors:** E. M. R. Critchley


### HYPOPHYSECTOMY FOR METASTATIC CARCINOMA OF THE BREAST:

### Some Electroencephalographic and Clinical Observations

E. M. R. CRITCHLEY

*From the EEG Department, Royal Free Hospital, Gray's Inn Road, London, W.C.1*

Received for publication June 5, 1964

Induction of a state of hypopituitarism is one of the measures that may be taken in the treatment of " hormone-sensitive " tumours to cause regression of the primary lesion or metastases, to alleviate bone pain and to secure some prolongation of life. Very rarely is it a primary treatment. The patient has usually been subjected already to a simple or radical mastectomy, deep X-ray therapy, oophorectomy either surgical or induced by irradiation, and received androgens or corticosteroids. Often the disease process has been long-standing, leaving the patient cachectic or dependent on regular doses of potent analgesics. The further treatment of these patients is difficult and after operation the patient has to be stabilised by replacement therapy with cortisone and thyroxine. (A recent review by Boesen (1964) explains the indications upon which hypophysectomies in this hospital have been based.)

In most centres hypophysectomy is preferred to adrenalectomy. Whereas in 1957 Atkins *et al.* found no statistical difference between the results of hypophysectomy and adrenalectomy, but noted a trend in favour of hypophysectomy, Pearson (1962), in selected patients, claimed a 90 per cent incidence of remissions compared with 50 per cent obtained by adrenalectomy.

Surgical hypophysectomy is a major undertaking, but implantations of radioactive isotopes into the pituitary fossa by the transnasal and transethmoidal routes have not yielded consistent results and have led to a high proportion of serious complications, notably cerebrospinal rhinorrhoea and meningitis and, less frequently, to irradiation damage to the optic nerves and chiasma (Bateman, 1962; Macbeth and Hall, 1962). The alternative, transcranial, approach has yet to be fully assessed. In general, and possibly more so with surgical than with irradiation hypophysectomy, cases with mammary, cutaneous, glandular, osseous and pulmonary lesions give the most rewarding results, whereas cases with cerebral and hepatic lesions do not (Falconer, 1963). If it can be shown that the success of surgical hypophysectomy is more likely to be influenced by the presence of intracranial metastases than in the case of implantation techniques, then the EEG could be of considerable value in the selection of the operative technique to be employed.

A retrospective study of the EEG in patients, treated either by subfrontal hypophysectomy or transcranial yttrium implantation, was therefore carried out, not only to detect the presence of pre-operative cerebral metastases, but also to ascertain the inter-relationships between operative and the presence of these metastases.

### METHODS

All the patients included in this report were operated on at the Royal Free Hospital between December 1959 and December 1962, Mr. E. J. Radley Smith performing the transcranial (subfrontal) hypophysectomies and Mr. A. M. H. Bennett the transcranial (stereotactic) yttrium implantations. The EEGs were taken, using 20 electrodes sited with reference to bony landmarks, approximately two to three weeks before operation and again three to six weeks post-operatively (average 3·9 weeks). In 6 patients a third EEG was recorded 3–6 months after operation, in one 20 months later and in 2 instances a fourth record was taken 3 years after operation. Thirty-one patients were studied in all. In 24 a subfrontal hypophysectomy was performed and in 7 transcerebral yttrium implantation.

The technique of subfrontal hypophysectomy is similar to that described by Murray A. Falconer (1963) but in addition 5 or 6 radioactive gold seeds are implanted into the fossa. Controlled hypothermia was used in 13 cases and in the others a general anaesthetic was given using the hyperventilation method to reduce the cerebral $pCO_2$. In all cases a heavy steroid cover is maintained over the operation period.

Yttrium implantation is performed under general anaesthesia. A 2 inch incision is made 2 inches to the right of and parallel to the sagittal plane of the vertex. A medium sized burr hole is made and the visceral dura cut. Bleeding points are diathermied and if a large cortical vessel is seen this, too, is diathermied. The incision is then closed with skin stitches and sprayed with Polybactrin. At this stage the stereotactic apparatus is fitted and the stylet (2 mm. diameter) inserted through the burr hole and passed transcerebrally into the pituitary fossa. Lateral and antero-posterior X-rays are taken for confirmation. On withdrawal it should not be possible to aspirate cerebrospinal fluid. The yttrium pieces are pushed down the tube and the attempted aspiration repeated. Finally the stylet is removed (Bennett, 1960).

Endocrine replacement therapy was begun with cortisone acetate 50 mg. *b.d.* one day before operation, increased over the period of the operation and thereafter reduced by 12·5 mg. or 25 mg. every third day until the patient was stabilised at 25 mg. *b.d.* In most patients L-thyroxine was started orally two weeks after operation. Although endocrine studies were performed, fine adjustment was not attempted.

### RESULTS AND CASE REPORTS

The detailed findings and salient features are summarised in the various Tables. Table I provides a rough comparison between the early EEGs and the initial assessment of the operation result, expressed schematically as :

$R^{++}$—definite regression of metastases.
$R^{+}$—some regression with symptomatic improvement.
O—no change.
W—worse.

Table II provides a quick analysis of the principal EEG changes and becomes important in the detailed discussion, and Table III outlines all 31 case histories.

In every patient in whom the pituitary was approached by the subfrontal route, slow or intermediate slow activity waves between 3–6 c/s were seen post-

TABLE I.—*Comparison of Immediate Pre- and Post-Operation EEGs with the Initial Assessment of the Operation Result*

| | Ages | Survival | Antecedent history | EEG abnormalities before operation | Operation result | EEG abnormalities after operation |
|---|---|---|---|---|---|---|
| *Hypophysectomy* | | | | | | |
| Alive 11 | range 47–65 years<br>average 50·1 years | 1–3 years | 1–14 years<br>5·6 years | 4 | $R^{++}$ 5<br>$R^{+}$ 5<br>O 1<br>Worse 0 | 5 |
| Dead 13 | range 35–61 years<br>average 50·6 years | 5–29 months<br>13·9 months | 1–16 years<br>3·9 years | 8 | $R^{++}$ 0<br>$R^{+}$ 5<br>O 5<br>Worse 3 | 10 |
| *Yttrium* | | | | | | |
| Alive 3 | range 46–61 years<br>average 54·3 years | 1–3 years | 2–11 years<br>6·9 years | 2 | $R^{++}$ 1<br>$R^{+}$ 2<br>O 0<br>Worse 0 | 2 |
| Dead 4 | range 55–71 years<br>average 60 years | 14–22 months<br>18 months | 2·5–27 years<br>9·1 years | 3 | $R^{++}$ 0<br>$R^{+}$ 4<br>O 0<br>Worse 0 | 3 |
| *Total* | | | | | | |
| 31 | range 35–71 years<br>average 52 years | 14·5 months<br>(dead only) | 5·5 years | 17 | $R^{++}$ 6<br>$R^{+}$ 16<br>O 6<br>Worse 3 | 20 |

operatively to a greater or lesser extent over the right frontal region. This activity was well formed in those EEGs taken three or four weeks after operation, less clearly seen at 6–7 weeks and had diminished considerably in the follow-up records at 3–6 months. Particularly where there was evidence of frontal lobe secondaries—suggested by the pre-operative EEG or seen at operation—and in those instances where a transient left hemiplegia occurred lasting up to a week after operation, this frontal asymmetry was more extensive, spreading into the central regions or even over the whole hemisphere. Sharp waves were frequently seen but it is virtually impossible to draw hard and fast conclusions from their presence. They have been tabulated in Table II and from this it will be seen that, though they are frequently associated with the presence of metastases, they are equally frequently seen in seemingly normal records.

*Case report I* (To illustrate the natural evolution of right frontal activity)

*E.P.* (14).—This patient, aged 65, first noticed a feeling of heaviness and discomfort in the left breast in December 1959, but did not seek medical advice until the following April when the nipple became retracted. When first seen, she had metastases in the lungs and bones, and an early hypophysectomy was advised. She has remained very well since the operation (June 1960 : hyperventilation anaesthesia) and in August 1963 was readmitted for a simple mastectomy.

TABLE II.—*An Analysis of the Principal EEG Changes Omitting Right Anterior Frontal Changes*

| Case | Number | Speed and stability of the alpha rhythm | | Theta > 10 per cent or presence of slow (delta) | | Abundance of fast activity | | Spikes or sharp waves | | Protentiation by hyperventilation | | Normal or raised $^{131}$I uptake | Diabetes insipidus | Subtotal removal | Parasellar secondaries | Cerebral secondaries (at operation) | Other comments |
|---|---|---|---|---|---|---|---|---|---|---|---|---|---|---|---|---|---|
| | | Pre | Post | Pre | Post | Pre | Post | Pre | Post | Pre | Post | | | | | | |
| JT | 1 | 9–10 | 8 | .. | + | .. | .. | .. | .. | + | + | .. | + | .. | .. | .. | |
| RM | 2 | US* | US | + | + | .. | .. | ++ | ++ | .. | + | .. | .. | .. | .. | .. | Toxic |
| VF | 3 | 12 | 12 | .. | .. | ++ | + | .. | .. | .. | .. | .. | + | .. | .. | .. | |
| JM | 4 | US | 8–10 | .. | .. | .. | + | + | .. | + | + | .. | .. | .. | .. | .. | |
| MA | 5 | 10–11 | 10–11 | .. | .. | .. | .. | .. | + | + | + | .. | .. | .. | .. | .. | |
| NB | 6 | 8–10 | 8–10 | .. | .. | + | .. | .. | + | .. | .. | .. | .. | .. | .. | .. | |
| IS | 7 | 10 | US | .. | + | .. | + | + | ++ | + | ++ | .. | + | .. | .. | .. | |
| MS | 8 | US | US | .. | .. | + | + | .. | .. | + | + | .. | .. | + | .. | .. | |
| BP | 9 | 8–10 | US | + | ++ | + | + | + | + | +++ | ++ | .. | + | .. | .. | .. | Addisons disease pre-op. |
| LL | 10 | 10–12 | 10–12 | .. | + | + | + | + | + | + | + | .. | + | .. | .. | .. | |
| EN | 11 | 10 | 10 | .. | .. | + | + | .. | + | .. | .. | .. | .. | + | + | .. | |
| AV | 12 | 12 | 13 | .. | + | + | ++ | .. | + | .. | .. | + | .. | .. | .. | + | |
| DC | 13 | 11–12 | 10–12 | .. | .. | + | .. | + | .. | + | + | .. | .. | .. | + | .. | |
| EP | 14 | 9–12 | 10 | .. | .. | + | + | + | + | .. | .. | .. | .. | .. | .. | .. | |
| VJ | 15 | US | US | .. | .. | + | .. | + | + | ++ | ++ | .. | + | .. | .. | + | Vertigo pre-op. |
| EMc | 16 | 10–12 | US | .. | .. | + | .. | + | .. | + | + | .. | .. | .. | .. | .. | |
| DT | 17 | 9–10 | 8–9 | .. | + | .. | .. | .. | + | .. | ++ | .. | + | + | .. | + | |
| CB | 18 | US | US | .. | .. | + | + | + | + | + | + | + | + | .. | + | .. | |
| OS | 19 | 10 | 9–10 | + | + | .. | + | + | .. | + | ++ | .. | + | + | .. | .. | |
| HB | 20 | US | US | ++ | + | ++ | .. | + | + | .. | + | .. | + | .. | .. | + | |
| HH | 21 | 10 | 10 | .. | .. | + | .. | .. | + | .. | + | + | .. | + | + | .. | |
| MF | 22 | 10 | 10 | .. | .. | .. | .. | .. | .. | + | + | .. | + | + | .. | .. | |
| DC | 23 | 10 | 10 | .. | .. | .. | .. | .. | .. | + + | + | .. | + | + | .. | .. | |
| MS | 24 | 10 | 9–10 | .. | .. | .. | .. | .. | .. | .. | .. | .. | + | .. | .. | .. | |
| HN | 25 | 8 | US | + | + | .. | .. | + | .. | ++ | ++ | .. | + | .. | .. | .. | |
| JL | 26 | 9–10 | 8–9 | .. | + | .. | .. | + | + | .. | + | .. | + | .. | .. | .. | Vascular Abn. |
| AJ | 27 | 9–10 | 8–9 | .. | + | .. | .. | .. | .. | .. | + | .. | + | .. | .. | .. | |
| RC | 28 | 10 | 10 | .. | .. | + | + | .. | .. | + | .. | + | .. | .. | .. | .. | Orbital secondaries. |
| DD | 29 | 9–10 | 8–10 | .. | .. | ++ | ++ | + | + | .. | + | .. | .. | + | + | .. | |
| AE | 30 | 10 | 9–10 | .. | + | + | + | .. | + | + | + | .. | .. | .. | .. | .. | |
| KF | 31 | US | US | + | .. | .. | .. | + | + | + | + | .. | + | .. | .. | .. | |

* US = Unstable.

EEGs were taken three weeks preceding operation, and 3 weeks, 6 months and 3 years afterwards. The right frontal activity was best seen on the first EEG after operation and subsequently showed a regression.

*Pre-operative EEG.*—" The alpha rhythm could be recognised at 9–12 c/s mixed with a considerable amount of generalised low amplitude faster activity. Some intermediate slow activity and a few sharp elements were occasionally seen, but there was no change during and after overbreathing. The responses to photic stimulation were regular and symmetrical."

*2nd EEG.*—" The alpha rhythm was regular and fairly symmetrical mixed with faster activities and well-blocked on eye-opening. Some irregular intermediate slow activity and slower waves were often seen over the anterior and

TABLE III.—

| Group | No. | Patient | Age | Menstrual state | Mastectomy | Deep X-ray therapy | Androgens | Other drugs | Antecedent history (years) | Survival (months) | Date of operation | Anaesthetic used | Excess bleeding | Incomplete removal | Diuresis 4 litres | Electrolyte disturbance | Hypoglycaemia | Immediate success | Time after op of 2nd EEG (weeks) |
|---|---|---|---|---|---|---|---|---|---|---|---|---|---|---|---|---|---|---|---|
| HYPOPHYSECTOMY (R. Subfrontal) (24 cases) | 1 | JT | 56 | N | + | + | .. | .. | 1 | 16 | 16–12–59 | c | .. | .. | + | .. | .. | O | 3 |
| | 2 | RM | 49 | S | .. | .. | + | + | 4 | 5 | 9–3–60 | c | .. | .. | .. | .. | .. | W | 3 |
| | 3 | VF | 55 | N | + | + | .. | .. | 7 | 16 | 11–5–60 | hv | .. | .. | + | .. | + | R+ | 4 |
| | 4 | JM | 52 | N | + | + | + | + | 3½ | 24 | 6–7–60 | c | .. | .. | + | .. | .. | O | 3 |
| | 5 | MA | 45 | S | .. | .. | + | .. | 1 | 12 | 13–7–60 | c | .. | .. | .. | .. | .. | R+ | 3½ |
| | 6 | NB | 50 | N | .. | + | + | + | 2½ | 15 | 3–8–60 | .. | .. | .. | .. | .. | .. | O | 4 |
| | 7 | IS | 45 | A | .. | + | + | + | 3 | 5 | 17–8–60 | .. | + | .. | + | .. | .. | W | 3½ |
| | 8 | MS | 61 | N | + | + | + | + | 16 | 8 | 21–9–60 | hv | .. | ST | .. | + | + | O | 4 |
| | 9 | BP | 35 | A | + | + | .. | + | 5 | 9 | 17–11–60 | hv | .. | .. | ++ | .. | .. | W | 6 |
| | 10 | LL | 54 | A | + | + | + | .. | 4 | 11 | 8–3–61 | c | .. | .. | + | .. | .. | R+ | 7 |
| | 11 | EN | 47 | A | + | + | .. | .. | 4 | s | 20–1–60 | c | ++ | ST | .. | + | .. | R++ | 4 |
| | 12 | AV | 51 | A | + | + | + | .. | 4 | s | 20–1–60 | c | .. | .. | .. | ++ | .. | R++ | 4 |
| | 13 | DC | 47 | .. | + | + | + | .. | 1 | s | 18–5–60 | .. | .. | .. | .. | .. | .. | R+ | 4 |
| | 14 | EP | 65 | N | .. | .. | .. | .. | 1 | s | 17–6–60 | hv | .. | .. | .. | .. | .. | R++ | 3 |
| | 15 | VJ | 59 | N | + | + | + | .. | 1 | 29 | 20–7–60 | hv | .. | .. | + | .. | .. | R+ | 3½ |
| | 16 | EM | 56 | A | + | .. | + | .. | 12 | s | 9–11–60 | c | .. | .. | .. | .. | .. | R+ | 4 |
| | 17 | DT | 54 | A | + | + | + | .. | 14 | s | 19–1–61 | hv | .. | ST | + | .. | .. | O | 6 |
| | 18 | CB | 65 | N | .. | .. | + | .. | 1 | s | 1–2–61 | c | .. | .. | + | .. | .. | R+ | 4 |
| | 19 | OS | 41 | S | + | + | .. | .. | 2 | 20 | 1–3–61 | c | ++ | ST | + | .. | .. | O | 4 |
| | 20 | HB | 51 | A | + | + | + | .. | 9 | s | 12–6–61 | c | .. | .. | + | .. | .. | R+ | 3 |
| | 21 | HH | 56 | N | .. | .. | .. | .. | 2 | 10 | 13–9–61 | hv | + | ST | .. | .. | .. | R+ | 4 |
| | 22 | MF | 38 | .. | + | + | + | + | 4 | s | 12–12–62 | hv | .. | ST | + | .. | .. | R+ | 2 |
| | 23 | DC | 41 | S | + | .. | .. | .. | 4 | s | 16–3–61 | c | ++ | ST | + | .. | .. | R++ | 4 |
| | 24 | MS | 46 | A | + | + | + | .. | 8 | s | 24–1–62 | c | .. | .. | + | .. | .. | R++ | 4 |
| YTTRIUM 90 (7 cases) | 25 | HN | 55 | N | + | + | .. | .. | 4 | 14 | 15–3–62 | ga | .. | .. | + | .. | .. | R+ | 4 |
| | 26 | JL | 71 | N | + | + | + | + | 27 | 22 | 7–9–60 | ga | .. | .. | + | .. | .. | R+ | 5 |
| | 27 | AJ | 55 | N | + | + | + | + | 2½ | 18 | 1–3–61 | ga | .. | .. | + | .. | .. | R+ | 1 |
| | 28 | RC | 46 | N | + | .. | + | .. | 4 | s | 15–11–61 | ga | .. | .. | .. | .. | .. | R+ | 6 |
| | 29 | DD | 59 | N | + | + | .. | + | 3 | 18 | 17–1–62 | ga | .. | ST | .. | .. | .. | R+ | 5 |
| | 30 | AE | 56 | N | + | + | + | .. | 11 | s | 3–8–61 | ga | .. | .. | .. | .. | .. | R+ | 4 |
| | 31 | KF | 61 | N | + | + | + | .. | 7 | s | 1–3–61 | ga | .. | .. | + | .. | .. | R++ | 4 |

Menstrual State : N—Natural menopause. S—Menstruating at time of operation. A—Artificial menopause.

Other Drugs : Prednisone (2), oestrogens (6), cytotoxic drugs (4).

Anaesthetic used : c— hypothermia. h.v.—hyperventilation. ga.—general anaesthesia, straightforward.

Immediate Success : R++—definite regression of metastases, R+—some regression with symptomatic improvement.
O—no change.
W—worse.

EEG summaries : Ant RF—anterior right frontal slow activity. LF—left frontal.
O—normal. 2″ possible secondaries. asym—asymmetry ? cause.
Mid R—middle of right hemisphere.

*Summary of Case-histories*

| Interval between 1st and 2nd EEG | Skull metastases | Cerebral metastases | Neurological signs pre- and post-op. | Summary of 1st EEG | Summary of 2nd EEG | Subsequent EEGs | |
|---|---|---|---|---|---|---|---|
| 4 | .. | .. | Loss of eye | L. temp ab | Ant RF L temp. ab. | .. | |
| 4 | .. | .. | .. | Toxic asym | Toxic worse | .. | P.M.: soft rt. frontal lobe |
| 16 | .. | .. | .. | O | Ant RF | .. | |
| 8 | .. | .. | .. | O | Ant RF | .. | |
| 6 | .. | .. | Irr. pupils | 2″ | Ant RF worse | (3/12) less RF asym | |
| 4 | .. | .. | .. | O | Ant RF | (3/12) less RF asym | |
| 5 | .. | .. | .. | Asym ? 2″ | Ant RF & asym | .. | |
| 5 | .. | + | .. | O | Ant RF & asym | .. | |
| 10 | + | + | L. paresis post-op. | 2″ RF | Worse | .. | Small brain at op., P.M.: soft R.F.L. |
| 5 | + | + | .. | Asym ? 2″ | Ant RF & asym | .. | P.M.: soft R.F.L. |
| 6 | + | .. | .. | O | Ant RF | .. | |
| 5 | .. | .. | .. | O | Ant RF & excess fast | .. | Exophth., hyper-thyroid, pigmented. |
| 5 | .. | .. | L. paresis post-op. | O | Ant RF | (6/12+3 yr) asym; 2″. | |
| 6 | .. | .. | .. | O | Ant RF | (5/12; 3 yr) less RF asym; 2″. | |
| 8 | .. | .. | .. | Asym ? 2″ | Ant RF & asym | .. | |
| 7 | .. | .. | .. | 2″ | Ant RF & asym | .. | |
| 6 | + | + | L. paresis post-op. | O | Ant RF & asym | .. | |
| 8 | .. | + | diplopia | Asym | Ant RF & asym | .. | Small brain at op., normal $^{131}I$ |
| 5½ | .. | .. | .. | Asym | Ant RF & LF | .. | |
| 5 | + | .. | .. | 2″ | Ant RF improvement | .. | |
| 6 | + | + | .. | O | Ant RF & asym | .. | P.M.: pituitary remnants brain bulging at op. |
| 5 | + | .. | .. | O | Ant RF & LF | .. | |
| 5 | .. | .. | .. | Asym | Ant RF & asym | .. | |
| 6 | .. | .. | .. | O | Ant RF | .. | |
| 6 | + | .. | .. | 2″ | 2″ no change | .. | |
| 6 | + | .. | .. | Vascular ab | No change | (20/12) no change | No macroscopic secondaries seen at P.M. |
| 9 | .. | .. | .. | O | Mid R sl.abn. | .. | |
| 35 | + | .. | L. ptosis | Asym? 2″ | Asym | (7/12) asym. sl. worse | Normal $^{131}I$ |
| 8 | + | + | .. | 2″ | 2″ | .. | |
| 5 | + | .. | .. | O | O | .. | |
| 6 | .. | .. | .. | Asym | Improvement | .. | |

middle thirds of the right hemisphere. During overbreathing there was little change and the responses to photic stimulation were regular and symmetrical."

*3rd EEG.*—" A rhythmic activity between 10–20 c/s was seen over the posterior half of the head, mixed with a moderate amount of intermediate slow activity. A few slower waves and infrequent sharp elements were seen in the left temporal and in the right fronto-central regions. During overbreathing there was a slight increase in irregular intermediate slow activity and a variable asymmetry persisted. The responses to photic stimulation were regular and fairly symmetrical."

*4th EEG.*—" Occasionally some unstable alpha rhythm could be seen mixed with a considerable amount of lower amplitude faster and slower elements. During overbreathing there was a generalised increase in activity, more obvious over the right than over the left frontocentral and temporal regions. The responses to photic stimulation were of low amplitude."

It is possible, as evidenced by the siting of the post-operative changes in the right frontal lobe and their gradual regression in the ensuing months, that these changes were the result of trauma due to the operation. However, other abnormalities, situated elsewhere and failing to resolve, need more detailed explanation.

In three patients a marked right-sided abnormality coincided with the development of a transient left hemiplegia. In two, intracranial metastases were seen at operation ; the operation notes of the third were regrettably lost. Posterior abnormalities on one or other side were seen in four patients. In three, involvement of the parasellar region by secondaries was seen at operation and in the fourth the diaphragma sella was found thickened at operation and subsequently at autopsy a parasellar secondary was discovered.

*Case report II* (Fig. 1)

*H.H.* (21).—This patient aged 56, underwent hypophysectomy with hyperventilation anaesthesia immediately after biopsy confirmation of a breast neoplasm believed to have been present for two years. X-rays had shown frontal osteolytic areas. The diaphragma sella was very thick and because of excessive bleeding the hypophysectomy was incomplete. However, six $^{198}$Au seeds were inserted into the fossa. The iodine 131 uptake remained normal but relief of bone pain was complete. Ten months later she was readmitted with malignant ascites and died.

*Autopsy findings : The brain* : the vertical surface of the frontal lobe showed superficial softening of the grey matter and, at one point, complete destruction of this layer. A zone of gliosis surrounded the affected tissue. *The pituitary fossa :* a large amount of anterior and posterior lobe tissue remained. In the pars anterior frequent small cysts were present and, although the normal range of anterior lobe cells was identified, they were much larger than usual. Tumour deposit was present in the pars nervosa and infiltrating the surrounding bone.

### *Yttrium Implantation*

As anticipated, EEG abnormalities resulting from operative trauma were considerably less ; only if it were necessary to coagulate a cortical vein would

FIG. 1.—EEGs of case 21, H.H.

one expect to see some indication of the operation site. Such evidence was present in only one patient, otherwise, in the series, no new features emerged save for a slight improvement noted in one record (K. F., 31). Yet 5 out of 7 showed X-ray evidence of skull metastases and intracranial metastases were suspected in at least 5.

*Case report III* (Fig. 2)

*J. L.* (26).—This patient, aged 71, had been treated successively with DXT, testosterone and thiotepa for a neoplasm first diagnosed 27 years earlier. In September 1960 yttrium implantation was performed for local recurrence and several osseous deposits. In July 1962 she was readmitted with severe headache and neck stiffness. The cerebrospinal fluid showed 15 W.B.C./c.mm., 80 mg. per cent protein and she responded to antibiotics, but on July 11th she had a myocardial infarct and on July 18th collapsed and died from pulmonary embolism. At autopsy no macroscopic evidence of malignancy was found.

FIG. 2.—Case 26, J. L. Pre-operative, 5 weeks post-operative and 20 months post-operative EEGs.

*Other Considerations*

This example is helpful in that it draws attention to one of the vast motley of factors which must be taken into consideration. Hypophysectomy entails complex endocrinological changes, affects a disease process which is already widespread, and is a difficult undertaking.

*The presence of pre-existing neurological and cerebrovascular abnormalities*

Besides J.L. there were a few patients with non-metastastic cranial abnormalities, unaffected by the operative changes. One patient J. T. (1) had had her left eye removed as a child and another, M. A. (5), was noted to have irregular pupils. Two patients had eye signs probably resulting from secondary deposits in the orbit. Such abnormalities have to be recognised but do not disturb the pattern of events.

*The type of anaesthetic used for the operation*

Hypothermia was used in 13 hypophysectomies and a general anaesthetic involving a hyperventilation technique was used for the remainder. Quite extensive operations are well documented in the literature using hypothermia without any deleterious effect on the EEG; thus Benvenuto *et al.* (1961) described excision of an extracranial aneurysm of the internal carotid artery employing hypothermia. The patient's body temperature was lowered to 30–35° C. and the internal carotid artery was occluded for 40 minutes. During this time the electroencephalogram remained normal. Post-operatively the angiogram showed occlusion of the external carotid artery at the site of the anastomosis. The patient showed no neurological deficit.

The technique of hyperventilation anaesthesia is aimed at reducing the $pCO_2$ and this, in turn, reduces the cerebral blood flow, cerebral venous $pO_2$ and possibly the cerebral tissue $pO_2$ (Sugioka and Davis, 1960). The resultant changes have only a minimal and transient effect on cerebral function (Allen and Morris, 1962). The more mundane post-operative disturbances in $pO_2$ and $pCO_2$ which might cause cerebral disturbances resulting from general surgical operations were discussed by M. Hobsley (1963) in his Hunterian Lecture. A physiological shunt can develop representing 7–10 per cent of the cardiac output with consequent defective oxygenation especially in bronchitic chests with a tendency to segmental collapse. In fact, in this series, there were no stormy post-operative complications and, by delaying the subsequent EEG to three weeks post-operatively the minor alterations were minimised.

*Complications at the time of operation*

Excessive bleeding occurred in 5 patients. As a drain was inserted into the pituitary fossa, haematoma formation can be considered unlikely. However, excessive bleeding was the commonest cause for the removal to be adjudged subtotal rather than complete.

One patient R. M. (2) became hypotensive at the end of the operation and in two others intravenous urea was used to reduce cerebral oedema. These complications did not appear to have resulted in any marked EEG changes.

*Post-operative complications*

*Diabetes insipidus.*—Polyuria and polydipsia was seen in 18 of the 31 patients. These figures accord with those of Murray Falconer (1963) and others after both transcranial and transnasal operations. "They appear usually within 2 weeks (generally within a few days) but they always disappear at some time within 3 months. They are probably due to injury of the pituitary stalk at the time of operation and therefore we try to alleviate this by cutting the pituitary stalk low down flush with the diaphragma. Possibly as a consequence of this precaution we now observe this complication in its troublesome form less frequently." This polyuria seldom reaches the proportions found in true diabetes insipidus. The urine volumes in those in whom the adenohypophysis has been removed is seldom more than 4–5 litres as compared with 6–10 litres/day where the adenohypophysis is intact. This has been attributed to the low rate of glomerular filtration resulting from growth hormone and adrenosteroid deficiency. Furthermore, hypopituitarism differs from adrenocortical insufficiency in that aldosterone excretion continues and actually increases if the patient is challenged with a low salt diet (Peters *et al.*, 1954).

The various endocrine factors which can affect diuresis are reviewed by

Maccubbin and Van Buren (1963) ; they have also histologically confirmed Murray Falconer's dictum that " severe polyuria can be avoided by cutting the neural stalk close to the diaphragma sella " by study of the natural evolution of the polyuria and regeneration of cells in the supra-optic and para-ventricular nuclei after stalk section at various heights.

It will be seen in the present series that the most severe polyuric disturbances were found in those cases with evidence of metastasic deposits in the parasellar region. This renders the EEG features of the disturbance difficult to interpret. Rohmer *et al.* (1959) report that, except for symptomatic diabetes insipidus due to tumours or following meningoencephalitis, normal EEG traces are the rule but an abundance of fast activity may occur. As an abundance of fast activity was frequently seen in those cases with parasellar tumours usually in the region of the vertex, both before and after operation, interpretation of an EEG " polyuric disturbance " is not possible and Table II has been prepared to show such chance correlation as can be observed.

*Hypoglycaemia and electrolyte disturbances.*—Transient hypoglycaemia was seen in two patients, but the tendency to develop hypoglycaemia can be corrected by cortisone administration. Thus, unless irreparable neurological damage has resulted from the hypoglycaemic episode, the effect of postoperative hypoglycaemia is unlikely to be seen in EEGs done three or four weeks later. Similarly, transient electrolyte disturbances were mostly corrected by cortisone replacement before the post-operative EEG. Only in one patient (B. P.), who had had a previous adrenalectomy and showed evidence of adrenocortical deficiency four weeks before operation which responded to additional cortisone supplements and fluorocortisone, is there suggestive evidence that endocrine replacement might not have corrected the resultant endocrine deficit. By and large, it proved relatively easy to achieve endocrine stability ; minute adjustments in dosage were not required and the EEG remained stable over a wide dose range.

*Completeness of hypophysectomy.*—Subfrontal hypophysectomy does not guarantee the complete removal of the pituitary gland but in almost all cases (to quote Daughaday, 1962) the amount of functioning pituitary tissue remaining in cases of hypopituitarism due to post partum necrosis or chromophobe adenoma exceeds that occurring after skilled pituitary surgery. Murray Falconer has estimated that fragments of the gland up to about 10 per cent of its volume are sometimes left behind. " A criticism of our operative technique is that we approach the pituitary gland at an angle of about 20° to the right of the median longitudinal plane, and also more or less tangentically to the diaphragma. Thus, after removal of the gland we cannot see the anterior or right-hand walls of the pituitary fossa, but have to rely upon our scraping techniques to remove fragments there. "

The additional insertion of radioactive gold seeds should further decrease the amount of remaining viable tissue but even so, as in the case of H. H., this cannot be guaranteed. For one reason or another nine patients were considered to have had a subtotal removal and four patients, not all included in this group, showed a normal or raised $^{131}$I uptake and two had endocrine exophthalmos after operation. There are several possible explanations for the survival of thyroid function after hypophysectomy :

(1) the obvious one, that extirpation was incomplete and regeneration of active tissue from residual fragments had occurred. A recent paper by Sprunt

*et al.* (1963) has emphasised that the routine biochemical tests now used to detect incomplete ablation after yttrium implantation are relatively insensitive.

(2) that small remnants of tissue derived from Rathke's pouch may persist into adult life within or just below the sphenoid bone. It is speculated that these cells may assume secretory activity after removal of the main body of the pituitary (Daughaday, 1962).

(3) the presence of an extrapituitary (and probably hypothalamic) thyroid stimulator. This has long been suggested by continental authors—Lhermitte 1928, Alajouanine 1932, Roche 1937, Poltzer 1938 and de Gennes *et al.* 1951—and Adams and McKenzie have managed to assay a long acting thyroid stimulator and have produced evidence highly suggestive of an extrahypophyseal site of origin of this hormone. In an addition to his 1961 paper, McKenzie writes : " Pituitary tissue has now been assayed. It was obtained at necropsy from a patient with Graves disease whose serum in life contained the thyroid activator. When the pituitary was subjected to extraction with acetone and homogenisation in water or 0·9 per cent NaCl solution, the resulting material contained only thyrotrophin and no thyroid activator by assay in mice. Munro *et al.* (1960) have made similar observations ".

(4) that toxic thyroid adenomata may function independently of pituitary control.

These comments on the completeness of the ablation are germane, for Bancaud *et al.* (1962) studying the EEGs in 36 patients in whom yttrium implantation by the transnasal route had been performed divided up their results in respect of the EEG changes previously described by Decourt *et al.* (1962) in adult anterior hypophyseal insufficiency—slowing of the basal rhythm, presence of theta and delta rhythms without any increase in amplitude, a poor blocking reaction and diminished response to light and hyperventilation—and identified two distinct biological pictures : total hypophysectomy, and partial hypophysectomy with persistent secretion of antidiuretic hormone. The two types were not rigid but were interchangeable, passing through short transitional periods. Nonetheless, 29 out of their 36 patients could be firmly categorised.

Those classed as " total hypophysectomy " appeared to have :

(1) an increase in the frequency of the alpha rhythm.
(2) no change in the blocking reaction or in their reaction to P.S., and
(3) theta rhythms seen in the central regions.

In contrast, those classed as " partial hypophysectomy " showed :
(a) an extinction of slow waves.
(b) a diminution in the frequency of the alpha rhythm, and
(c) a clear improvement in the alpha blocking reaction and in the reaction to P.S.

The present study fails to reveal a similar division. The results do not point to a clear delineation of categories, and it was considered better to specify markedly unstable rhythms as such, rather than attempt a definitiveness which did not exist.

### DISCUSSION

The above considerations on examination have proved to be extraneous to the main theme (the EEG study of the inter-relationship of intracranial metastases and operation trauma) and, therefore, do not necessitate a reappraisal of the basic results. However, the post mortem study of cerebral sections for metastases has only been possible in a small proportion of the patients and considerable reliance has had to be placed on EEG and operation findings suggestive of intracranial metastases. Nevertheless, when comparing the value of palliative operative procedures in the presence of metastases, it is not the presence of the tumours *per se* but their effect on vital structures which is of ultimate concern. These are the very changes which the EEG tends to reflect. As early as 1936 Walter had shown that tumours, in themselves, do not give rise to abnormal electrical activity, this was a function of damaged neurones in the neighbourhood of tumours, as a result of haemorrhage, oedema, pressure, or of defects in their blood supply or metabolism. Thus a normal EEG does not exclude the presence of metastases but it does suggest that such metastases are either small or for the time being relatively harmless.

The occurrence, *de novo*, of intracranial metastases might have accounted for the development of the posterior anomalies seen in four patients, but the intervals between the first and second EEGs (averaging 7·5 weeks) is relatively short and uncomplicated metastastic growth is unlikely to explain the differences over this period. In contrast the EEGs were more widely spaced in the 7 cases which underwent yttrium implantation, but the operative trauma was considerably less and little change was detectable in the EEGs. When studying the frontal changes in the subfrontal hypophysectomies there appears to be a clear division between :

(1) those with normal preoperative EEGs in whom the operative trauma—elevation of the frontal lobe, incision of the diaphragma sellae and removal of the pituitary—produced a disturbance limited to the right frontal lobe and which gradually regressed in the ensuing months ; other areas being unaffected and the frontal lobe appearing firm at autopsy, and

(2) those where EEG disturbances suggested the presence of metastases before operation and subsequently developed a more gross EEG disturbance and, in some, the frontal lobe was found softened at autopsy. A common history was that the skull X-ray had shown osteolytic deposits in the frontal bones and at operation these metastases appeared to be infiltrating the brain ; the first EEG suggesting the presence of frontal abnormalities and the postoperative records showing widespread 3–6 c/s activity which failed to regress.

The trauma due to the operation in the two groups has been similar and the EEG features seen before operation appeared to point the subsequent pattern of events. With yttrium implantation studies there was little change in the EEG as might be expected from the less traumatic nature of the procedure but in five out of the seven cases there had been X-ray evidence of skull metastases and intracranial metastases were suspected in at least five.

From these observations it can be deduced that the behaviour of cerebral metastases is not uniform but fundamentally depends on the trauma to which the brain may be subjected. Thus the EEG comes to assume greater importance as a monitoring device for cerebral secondaries before deciding upon operation. Where cerebral secondaries are seen yttrium implantation may be preferred to surgical hypophysectomy or, alternatively, cytotoxic drugs might be preferred to " hormone surgery ".

### SUMMARY AND CONCLUSIONS

In this series of 31 cases there were a relatively large number of subfrontal hypophysectomies (24) as compared with 7 yttrium implantations. Subfrontal hypophysectomy is a major operation requiring prolonged anaesthesia and involving some surgical trauma.

Study of the EEG suggests that where there are no cerebral secondaries the operation is in most instances well withstood. Hypothermia and hyperventilation anaesthetic techniques do not cause prolonged disturbances of cerebral function and the traumatic changes in the frontal lobe gradually regress. If there are cerebral secondaries, operative trauma is liable to cause electroencephalographic changes in their neighbourhood and these do not regress in the postopertive period. Parasellar secondaries were relatively frequently encountered (4 out of 31 cases), often accompanied by an abundance of fast activity at or near the vertex. When these were removed a more severe polyuria than in the rest of the series was encountered (perhaps because of damage to the neural stalk nearer the supra-optic nucleus) and the fast activity often remained.

The present study has shown that transcerebral yttrium implantation can be performed without EEG alterations even when cerebral metastases are present.

A good correlation in both hypophysectomies and implantation operations was found between the EEG assessment and the immediate operative result.

I wish to thank Dr. G. Pampiglione, Consultant in Neurophysiology and Electroencephalography at this hospital, for suggesting this study and for help, advice and encouragement; Mr. E. J. Radley Smith, for his help and for permission to use the case reports of patients in his care; and Dr. H. E. Dimsdale, Mr. A. M. H. Bennett and Dr. E. Boesen for their help and advice.

### REFERENCES

Adams, D. D.—(1958) *J. clin. Endocrin.*, **18**, 699.
Alajouanine, T.—(1932) *Bull. Mém. Soc. méd. Hôp. Paris*, 1703.
Allen, G. D. and Morris, L. E.—(1962) *Brit. J. Anaesth.*, **34**, 296.
Atkins, H. J. B., Falconer, M. A., Hayward, J. L. and MacLean, K. S.—(1957) *Lancet*, i, 489.
Bancaud, J., Schaub, C., Talairach, J., Szikler, G., Tournoux, P., Bonis, A. and Marchand, H.—(1962) *Electroenceph. clin. Neurophysiol.*, **14**, 957.
Bateman, G. H.—(1962) *J. Laryng.*, **76**, 442.
Bennett, A. M. H.—(1960) *Brit. J. Radiol.*, **33**, 343.
Benvenuto, R., Depres, J. P., Pribam, H. F. W. and Callaghan, J. C.—(1961) *J. cardiovasc. Surg.*, **2**, 165.
Boesen, E.—(1964) Chapter 16, 'Recent Advances in Medicine', 14th edition. Ed. Beaumont and Dodds. London (J. & A. Churchill).
Daughaday. W. H.—(1962) 'Textbook of Endocrinology', 3rd edition. Ed. R. H. Williams. London (W. B. Saunders) pp. 12, 42.
Decourt, J., Michard, J-P., Brimani, Dj., and Dreyfus-Brisac, C.—(1962) *Ann. Endocr., Paris*, **23**, 637.
Falconer, M. A.—(1957) *Proc. Roy. Soc. Med.*, **50**, 861—(1963) *Ibid.*, **56**, 391.
de Gennes, L., Bricaire, H., Benzecry, I. and Villiaumey, J.—(1951) *Pr. méd.*, **59**, 41.
Hobsley, M.—(1963) *Ann. Roy. Coll. Surg. Engl.*, **33**, 105.
Lhermitte, J.—(1928) *Rev. neurol., Paris*, **35**, 125.

MACBETH, R. G. AND HALL, M.—(1962) *Arch. Otolaryng., Chicago*, **75**, 440.
MACCUBBIN, D. A. AND VAN BUREN, J. M.—(1963) *Brain*, **86**, 443.
McKENZIE, J. M.—(1958) *Endocrinology*, **62**, 865.—(1961) *Clin. Res.*, **9**, 336.—(1961) *J. clin. Endocrin.*, **21**, 635.—(1962) *Proc. Roy. Soc. Med.*, **55**, 539.
MUNRO, D. S., KILPATRICK, R., MAJOR, R. AND WILSON, G. M.—(1960) Abstract 599, 1st International Congress of Endocrinology, Copenhagen.
PEARSON, O. H.—(1962) ' Textbook of Endocrinology ', 3rd edition. Ed. R. H. Williams. London (W. B. Saunders), p. 938.
PETERS, J. P., GERMAN, W. J., MAN, E. B. AND WELT, L. G.—(1954) *Metabolism*, **3**, 118.
POLTZER, K.—(1938) *Wien. klin. Wschr.*, 560.
ROCHE, A.—(1937) *Pr. méd.*, **45**, 1157.
ROHMER, F., WACKENHEIM, A. AND KURTZ, D.—(1959) *Rev. neurol., Paris*, **100**, 297.
SPRUNT, J. G., BROWNIE, A. C. AND KINNEAR, J. S.—(1963) *Brit. med. J.*, ii, 1375.
SUGIOKA, K. AND DAVIS, D. A.—(1960) *Anesthesiology*, **21**, 135.
WALTER, W. G.—(1936) *Lancet*, ii, 305.